# Effects of *Theileria orientalis* Infection on Health Status and Productivity of Dairy Cows Reared inside Barns

**DOI:** 10.3390/pathogens10060650

**Published:** 2021-05-24

**Authors:** Yuki Fukushima, Tomoya Minamino, Yoko Mikurino, Kazuyuki Honkawa, Yoichiro Horii, Takako Taniguchi, Hirohisa Mekata, Yosuke Sasaki

**Affiliations:** 1 Course of Animal and Grassland Sciences, Graduate School of Agriculture, University of Miyazaki, Miyazaki 889-2192, Japan; ge16043@student.miyazaki-u.ac.jp; 2 Honkawa Ranch, Oita 877-0056, Japan; t-minamino@honkawa.jp (T.M.); mikurino@honkawa.jp (Y.M.); kazuyuki@honkawa.jp (K.H.); horii@honkawa.jp (Y.H.); 3 Center for Animal Disease Control, University of Miyazaki, Miyazaki 889-2192, Japan; t_iwata@cc.miyazaki-u.ac.jp (T.T.); mekata@cc.miyazaki-u.ac.jp (H.M.); 4 Department of Animal and Grassland Sciences, Faculty of Agriculture, University of Miyazaki, Miyazaki 889-2192, Japan

**Keywords:** epidemiology, health status, productivity, *Theileria orientalis* infection

## Abstract

The objective of the present study was to investigate the effects of *Theileria orientalis* on the severity of anemia, the prevalence of disease within 21 days after calving and productivity in cows raised inside barns. This longitudinal observational study, which was conducted on a commercial dairy farm in Japan, involved 627 Holstein cows subjected to PCR analysis for *T. orientalis*. In study 1, we collected blood samples from 156 sick cows within 21 days after calving, and we found the prevalence of *T. orientalis* infection to be 65.4%. In study 2, we randomly selected 471 cows during the dry period and collected blood samples to conduct PCR analysis for *T. orientalis* and determined the prevalence of *T. orientalis* infection to be 69.0%. Compared with the values for the *T. orientalis*-uninfected group, the *T. orientalis*-infected cows had significantly decreased hemoglobin concentrations and hematocrit, but there were no differences in the other complete blood count indexes between the two groups. In addition, there were no differences in productivity and the prevalence of major diseases between the *T. orientalis*-infected and uninfected cows. In summary, *T. orientalis* had few effects on anemia, productivity and the health of cows raised inside a barn.

## 1. Introduction

*Theileria orientalis*, which is a tick-borne protozoan parasite that causes mild to severe anemia in infected cattle [1], is distributed throughout many countries, including Australia, Britain, Iran, Japan, USA, Korea, and Russia [2]. Recently, New Zealand and Australia suffered serious epidemics of bovine anemia associated with *T. orientalis* [3,4,5]. Several studies carried out in Hokkaido and Kyushu, Japan, revealed that *T. orientalis* infections are a potentially serious problem in grazing cattle [6,7]. In Japan, ticks are the main causative agents of horizontal transmission of *T. orientalis*; however, approximately 10% of infections are due to vertical transmission from cows infected with *T. orientalis* to their calves [8]. Cattle appear to harbor the parasite for the remainder of their life after the initial infection [9,10]. However, few studies on *T. orientalis* have focused on cows raised inside barns. The outcomes of *T. orientalis* infections are highly dependent on environmental stress factors [11,12,13], and it is assumed that the protozoan replicates rapidly to cause symptoms, including fever and anemia, when the host is under any stress. The peripartum period is the most stressful for cows, and disease status during this period may progress depending on stress levels.

Recent studies have shown the negative impacts of *T. orientalis* infection on the milk production and fertility of grazing dairy cows [14,15,16]. Nevertheless, few studies have focused on the effects of *T. orientalis* on the productivity of cows raised inside barns. The objective of the present study was to investigate the effects of *T. orientalis* on the degree of anemia with peripartum diseases, the prevalence of diseases within 21 days after calving, and productivity (305-day milk yield and number of days from calving to conception) in cows raised inside a barn in Japan.

## 2. Results

**Figure 1:**
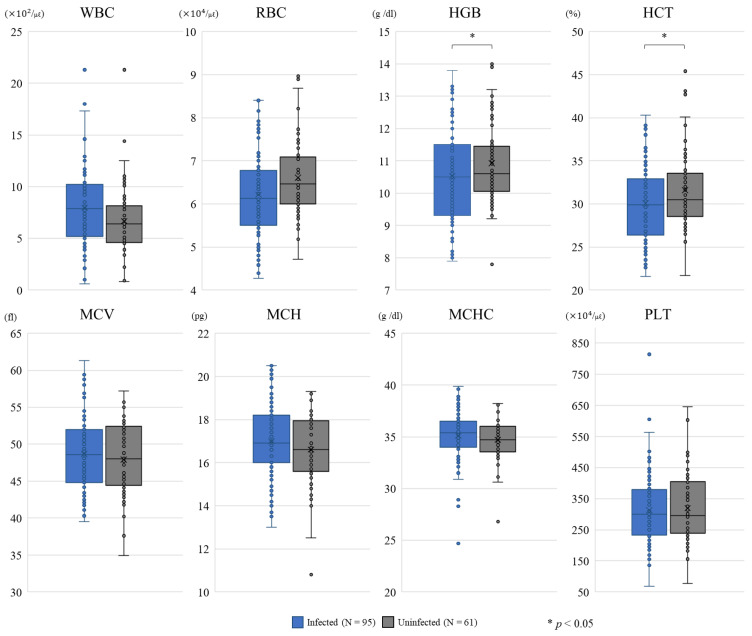
Comparison of complete blood count (CBC) indexes between cows infected and uninfected with *T. orientalis*. The CBC indices were red blood cell (RBC) counts, hemoglobin concentration (HGB), hematocrit (HCT), mean corpuscular volume (MCV), mean corpuscular hemoglobin (MCH), and mean corpuscular hemoglobin concentration (MCHC). White blood cells (WBC) and platelets (PLT) were also assessed.

Study 1 aimed to compare complete blood count (CBC) indices between the *T. orientalis*-infected and -uninfected cows in the 156 cows that showed clinical signs and were diagnosed as a sick animal within 21 days after calving. Out of 156 cows, the prevalence of *T. orientalis* was 60.9% (95/156). Comparisons of the CBC indices between the *T. orientalis*-infected and -uninfected cows are shown in Figure 1. Compared with the values for the *T. orientalis*-uninfected group, the *T. orientalis*-infected cows had significantly different CBC indices (*p* < 0.05), i.e., hemoglobin concentration (HGB) was decreased by 0.4 g/dL and hematocrit (HCT) was decreased by 1.5%. However, there were no significant differences in white blood cells (WBC), red blood cells (RBC), mean corpuscular volume (MCV), mean corpuscular hemoglobin (MCH), mean corpuscular hemoglobin concentration (MCHC), or platelets (PLT) between the *T. orientalis*-infected and -uninfected cows. Parity groups and origin of replacement cows were associated with CBC indices (*p* < 0.05), but there was no interaction between *T. orientalis* status and parity groups or origin of replacement cows on CBC indices.

Study 2 aimed to compare the prevalence of disease and productivity between the *T. orientalis*-infected and -uninfected cows in the 471 cows that underwent PCR analysis for *T. orientalis* during the dry period. Out of 471 cows, the prevalence of *T. orientalis* was 69.0% (325/471). Comparisons of the prevalence of each disease within 21 days after calving between the *T. orientalis*-infected (856 calving records in 325 cows) and -uninfected cows (317 calving records in 146 cows) are shown in Table 1. There were no differences in the prevalence of clinical mastitis, peracute mastitis, metabolic disorder, or peripartum disorder between the *T. orientalis*-infected and -uninfected cows. In addition, there were no differences in 305-day milk yield or number of days from calving to conception between the *T. orientalis*-infected and -uninfected cows (Table 2). Parity groups and origin of replacement cows were associated with 305-day milk yield, number of days from calving to conception, and the prevalence of peripartum disorder (*p* < 0.05), but there was no interaction between *T. orientalis* status and parity groups or origin of replacement cows on these variables.

## 3. Discussion

This is the first study to investigate the effects of *T. orientalis* on productivity in cows raised inside a barn. The present study showed that approximately 70% of the cows were infected with *T. orientalis*, which is higher than seen in previous studies on the prevalence of *T. orientalis* in grazing cattle [6,7,17], probably because we employed molecular diagnosis in this study. Cows are persistently infected during their lifetime after they contract *T. orientalis* [9,10], and the vertical transmission of *T. orientalis* occurs in approximately 10% of field cattle [8,18]. Therefore, the high prevalence of *T. orientalis* may be caused by the introduction of *T. orientalis*-infected cows from Australia or vertical transmission from *T. orientalis*-infected cows. Our results showed no effect of *T. orientalis* on productivity, fertility, or health, which differs from previous studies [14,15,16] conducted on grassland-raised cows. These findings indicate that the negative impacts of *T. orientalis* on barn-raised cows can be minimized by adopting good management procedures. For example, inside a barn, farmers can conduct more current nutritional management and intensive care and identify sick individuals earlier because of the ease of daily observation, and cows are under less stress from weather conditions compared with those on grassland, making such management procedures more effective for cattle maintained in barns than grassland.

Our results showed that there was no difference in the prevalence of major diseases within 21 days after calving between the *T. orientalis*-infected and -uninfected cows, indicating that cows infected with *T. orientalis* did not incur health problems under free barn conditions. Additionally, among the population of cows that showed clinical signs of disease and were diagnosed as a sick animal within 21 days after calving, we observed lower HGB and HCT in cows infected with *T. orientalis* than uninfected cows, but the differences were far smaller than those of cows raised on grassland [7,19,20]. Furthermore, there were no differences in the other CBC indices between the *T. orientalis*-infected and -uninfected cows. These results indicate that intensive management systems can inhibit the progress of anemia in cows infected with *T. orientalis*. In regard to the pathogenicity of *T. orientalis*, the Ikeda and Chitose genotypes are thought to be related to severity of the disease [21]. Both genotypes have already been confirmed on this farm [8]. Therefore, it is unlikely that a monopoly of nonpathogenic subtypes of *T. orientalis* biased the present results from this farm.

We found no differences in the 305-day milk yield and number of days from calving to conception between the *T. orientalis*-infected and -uninfected cows, which contrasts with previous studies that showed negative effects of *T. orientalis* on milk yield [15,16] and fertility [14]. Productivity loss in cows infected with *T. orientalis* is reportedly due to an increase in schizogony [16], which decreases nutritional condition and metabolic status. In the present study, cows had access to sufficient amounts of feed under barn conditions, which resulted in no difference in productivity.

The present study had several limitations that should be noted when interpreting the results. First, this study involved the analysis of data from one commercial farm, and the results cannot be generalized to all farms that maintain herds within barns. Second, other possible variables, such as nutritional condition and management issues, which we could not evaluate in this study, may have influenced the results. Nevertheless, the value of the present study was that the effects of *T. orientalis* on productivity and the health status of cows raised inside a barn were evaluated for the first time, and further studies analyzing more data are warranted to improve our understanding of this subject.

In conclusion, approximately 70% of cows were infected with *T. orientalis* despite being raised inside a barn, but *T. orientalis* had no effect on productivity or health status. Therefore, the negative impacts of *T. orientalis* can be minimized by adopting management procedures for barn-maintained cows.

## 4. Materials and Methods

### 4.1. Data Collection

Data from the present study were collected from a large dairy farm in Oita prefecture, Japan. Oita prefecture, which is located on the northern coast of Kyushu, has a temperate climate. The studied farm contained approximately 2500 Holstein cows, which were either purchased from Australia or home-produced and reared on the farm. All animals in this study were reared in an intensive system in which they were housed in a free barns. Sawdust was laid in the barn for the duration of their stay. The sawdust in each barn was changed once every 3 days and the manure was removed once every 2 days. No grazing was performed on this farm. Fixed-time artificial insemination was implemented in all cows after estrus synchronization, which was performed at approximately 40–50 days post-calving. Cows were bred by natural insemination if they failed to conceive at first insemination. In order to reduce heat stress, International Cooling Elements (I.C.E., Cargill Japan, Tokyo, Japan) and orally-administered sodium bicarbonate were fed to cows, and fans and water spray were used during the period from June to September.

### 4.2. Data Collection

A longitudinal observational study focusing on 627 Holstein cows was conducted in 2017–2020, involving PCR analysis for *T. orientalis*. In study 1, we collected blood samples from 156 cows that showed clinical signs and were diagnosed as a sick animal by a veterinarian within 21 days after calving. Blood records (n = 156) were used for analysis in study 1. In study 2, we randomly selected 471 cows during the dry period and collected blood samples to conduct PCR analysis for *T. orientalis*. For these 471 cows, we collected data on the cows, production records, and their health records. Information on the cows and production records were obtained from recording software used at the studied farm, and included identification number, parity, calving date, 305-day milk yield, conception date, and origin of replacement cows. Health records for the 21 days after calving were obtained from the clinical veterinary service section of the farm. The top and low 1% records of milk yield and number of days from calving to conception were excluded from our analysis as outliers. Thus, 1173 calving records were obtained and used for analysis in study 2.

As the data were obtained from a regional database and no experiments were performed on live animals, we did not seek University Animal Care and Use Committee approval. The study did not require any administrative permission to access the raw data from the regional database. On this farm, blood collections have been routinely done for clinical diagnosis and for hygiene control and the results of blood examination including CBC, biochemical examination, molecular examination, etc., have been collectively recorded as an individual animal’s history.

### 4.3. CBC Index and PCR Analysis for T. orientalis

The collected samples mentioned above were used to assess CBC indices. The CBC indices used were as follows: RBC counts, HGB, HCT, MCV, MCH, and MCHC. WBC and PLT were also assessed. These variables were determined using a particle counter (PCE-210N; ERMA Inc., Tokyo, Japan).

The blood samples were assessed using blood direct PCR to detect the *T. orientalis* p23 gene. To confirm *T. orientalis* infection, genomic DNA was extracted from the blood of the PCR-positive cattle and *T. orientalis* MPSP gene targeting universal region; Ikeda and Chitose genotypes were used for real-time PCR [22]. The method is described in our previous report [8]. On the studied farm, both genotypes were confirmed.

### 4.4. Definition and Categorization

Disease status was defined through the diagnosis and treatment of cows by the clinical veterinarians on this farm. Farm staff checked each cow’s condition every morning. If they found any clinical signs, a blood test was performed. Then, once the cow’s disease was confirmed, they were treated by clinical veterinarians. Disease status was classified into five groups: clinical mastitis, peracute mastitis, metabolic disorders, peripartum disorders and miscellaneous. The prevalence of each four major disease types was assessed by calculating the number of cows with each disease divided by the number of calvings. Peracute mastitis was defined as a cow that was diagnosed with mastitis and had a WBC less than 4000 cells/µL, and the other cows diagnosed with mastitis were classified as having clinical mastitis. We categorized the cows’ origin of replacement into two groups: cows produced on the farm or introduced from Australia. We classified parity into two groups, 1 and ≥2.

### 4.5. Statistical Analysis

Study 1 was conducted to compare CBC indices between *T. orientalis*-infected and -uninfected cows using a mixed-effects linear model. The observation unit was one cow. The dependent variables were the above-mentioned CBC indices, and the independent variables were *T. orientalis* status (whether a cow had *T. orientalis* (1 or 0) during the study period), parity, and whether the cows were produced on the farm or introduced from Australia. All possible two-way interactions between significant factors were included in all the models, but insignificant interactions (*p* > 0.05) were removed from the final models. Month of blood collection was included as a random effect.

Study 2 was designed to compare the prevalence of each disease within 21 days after calving and productivity between *T. orientalis*-infected and -uninfected cows using a mixed-effects logistic regression model or mixed-effects linear model. The dependent variables were disease status (a cow with or without (1 or 0) of each disease within 21 days after calving), 305-day milk yield, and number of days from calving to conception. The independent variables were *T. orientalis* status (whether a cow had *T. orientalis* (1 or 0) during the study period), parity, and whether the cows were produced on the farm or introduced from Australia. All possible two-way interactions between significant factors were included in all the models, but insignificant interactions (*p* > 0.05) were removed from the final models. Cows and calving year were included as random effects.

All statistical analyses were performed using SAS software version 9.4 (SAS Institute Inc., Cary, NC, USA). In each model, *p*-values less than 0.05 were considered significant.

## Figures and Tables

**Table 1:** Comparison of prevalence of major diseases within 21 days after calving between cows infected and uninfected with *T. orientalis*.

Disease Type	Uninfected Cows (N = 317)	Infected Cows (N = 856)	*p*-Value
	Prevalence	Prevalence	
Clinical mastitis	5.0	6.4	0.24
Peracute mastitis	1.6	0.8	0.22
Metabolic disorder	0.9	1.8	0.48
Peripartum disorder	4.7	2.9	0.69

**Table 2:** Comparison of productivity between cows infected and uninfected with *T. orientalis*.

Productivity	Uninfected Cows (N = 317)	Infected Cows (N = 856)	*p*-Value
	Mean ± SEM	Mean ± SEM	
305-day milk yield, kg	9650 ± 102.7	10,066 ± 67.0	0.11
Number of days from calving to conception	104.2 ± 3.4	110.9 ± 2.2	0.40

## Data Availability

The data presented in this study are available on request from the corresponding author. The data are not publicly available due to privacy.
